# Wuhan strain of SARS-CoV-2 triggers activation of immune evasion machinery similar to the one operated by cancer cells

**DOI:** 10.3389/fimmu.2025.1599352

**Published:** 2025-06-18

**Authors:** Maryam Abooali, Inna M. Yasinska, Gauri Thapa, Xi Lei, Kelly A. S. da Costa, Stephanie Schlichtner, Steffen M. Berger, Elizaveta Fasler-Kan, Nigel J. Temperton, Romina Vuono, Vadim V. Sumbayev

**Affiliations:** ^1^ Medway School of Pharmacy, Universities of Kent and Greenwich, Chatham, United Kingdom; ^2^ DKFZ-Hector Cancer Institute at the University Medical Center Mannheim, Mannheim, Germany; ^3^ Division of Personalized Medical Oncology (A420), German Cancer Research Center (DKFZ), Heidelberg, Germany; ^4^ Department of Personalized Oncology, University Hospital Mannheim, Medical Faculty Mannheim, University of Heidelberg, Mannheim, Germany; ^5^ Department of Pediatric Surgery, Children’s Hospital, Inselspital Bern, University of Bern and Department of Biomedical Research, University of Bern, Bern, Switzerland

**Keywords:** SARS-CoV-2, COVID-19, cancer, immune checkpoints, immune evasion

## Abstract

In the last 2 years, there has been an increasing concern that SARS-CoV-2 infection may represent a marker of undiagnosed cancers. A potential connection between COVID-19/long COVID and malignant transformation/cancer progression was reported in a number of studies. It is, however, unclear if the virus itself can cause malignant transformation or if it has a potential to support malignant processes in human body. We analyzed nasopharyngeal swabs collected from individuals infected with Wuhan strain of SARS-CoV-2 and conducted *in vitro* studies using BEAS-2B human bronchial epithelial cells. Here we report that Wuhan strain of SARS-CoV-2 and its spike protein induce activation of hypoxia-inducible factor 1 (HIF-1) transcription complex in infected cells. This effect is achieved through conversion of cellular 2-oxoglutarate into 2-hydroxy-glutarate, which most likely blocks the activity of HIF-1α prolyl hydroxylation. As such, it leads to activation of HIF-1, which triggers production of transforming growth factor–β type 1 (TGF-β). TGF-β induces expression of immune checkpoint proteins, such as galectin-9, programmed death-ligand 1, and indoleamine-2,3-dioxygenase, an enzyme, which is involved in production of immunosuppressive amino acid called L-kynurenine. These immune checkpoint pathways were capable of suppressing both helper and cytotoxic activities of T lymphocytes and, as such, could potentially support malignant processes in infected tissues.

## Introduction

COVID-19 pandemic has significantly affected and keeps affecting human populations worldwide. Despite the immune responses to COVID-19 infection, and long COVID reactions having now been extensively studied (1), there are still a large number of SARS-CoV-2 effects that remain to be understood. Recent evidence has clearly demonstrated potential connection of COVID-19 infection/long COVID and malignant transformation/cancer progression (2, 3). Several studies have revealed that cancer could be a potential long-term complication of COVID-19 infection, whereas other studies have shown that SARS-CoV-2 infection may represent a marker of undiagnosed cancers (4, 5). However, it is still unclear if the virus itself can cause malignant transformation or if it has a potential to support malignant processes in human body.

Immune evasion machinery operated by human embryonic and cancer cells includes the use of checkpoint immunosuppressive proteins, such as galectin-9, programmed death-ligand 1 (PD-L1), and indoleamine-2,3-dioxygenase (IDO) to suppress activities of T helpers, natural killer (NK), and cytotoxic T cells (6, 7). As a result, T helpers lose their ability to activate cytotoxic T and NK cells, as well as cytotoxic T cells become incapable of attacking and killing target cells.

Earlier studies have demonstrated that SARS-CoV-2 infection leads to elevated levels of galectin-9 and PD-L1 in blood plasma of patients; however, the mechanism, underlying these effects and sources of these immune checkpoint proteins remain unclear (8–11).

We were interested to uncover if COVID-19 (Wuhan ancestral strain, which caused the most severe complications, and potentially led to an increase in cancer case numbers (1–5)) infection can cause activation of immune evasion machinery, similar to the one operated by cancer cells. Activation of immune evasion machinery in affected tissues may significantly support the process of malignant transformation and tumor growth in these tissues helping the malignancies to escape the patient’s immune surveillance. Understanding these effects and underlying biochemical mechanisms was the aim of our work.

Here, we report, for the first time, that Wuhan strain of SARS-CoV-2 and its spike protein (S) induce activation of hypoxia-inducible factor 1 (HIF-1) transcription complex possibly by triggering conversion of cellular 2 oxoglutarate into 2-hydroxy-glutarate, which most likely blocks prolyl hydroxylation/degradation of inducible alpha subunit of HIF-1 (HIF-1α). Active HIF-1 induces production of transforming growth factor–β type 1 (TGF-β), which triggers expression of immune checkpoint proteins, such as galectin-9, PD-L1, and IDO1. These immune checkpoint pathways are capable of suppressing both helper and cytotoxic activities of T lymphocytes.

## Materials and methods

### Materials

Cell culture media, fetal bovine serum, supplements, and basic laboratory chemicals were obtained from Sigma (Suffolk, UK). Microtiter plates for enzyme-linked immunosorbent assay (ELISA) were obtained from Nunc (Roskilde, Denmark). Rabbit antibodies against galectin-9 (ab69630), phospho-S423/S425-Smad3 (ab52903), HIF-1α (ab51608), receptor for advanced glycation end products (RAGE) (ab3611), and granzyme B (ab134933) as well as mouse antibody against HIF-1α (ab1) were purchased from Abcam (Cambridge, UK). Mouse antibody against β-actin was purchased from Proteintech (66009-1-Ig, Manchester, UK). Goat anti-mouse and anti-rabbit fluorescently labeled dye secondary antibodies were obtained from LI-COR (Lincoln, Nebraska USA). ELISA-based assay kits for the detection of TGF-β, galectin-9, PD-L1, and Interleukin-2 (IL-2) and were purchased from Bio-Techne (R&D Systems, Abingdon, UK). All other chemicals used in this study were of the highest grade of purity and commercially available.

### Cell lines

Cell lines used in this work were purchased from either European Collection of Cell Cultures or American Type Culture Collection (ATCC). Cell lines were accompanied by authentication test certificates. BEAS-2B normal bronchial epithelium cells and LN18 human glioblastoma cells were cultured using Dulbecco's Modified Eagle Medium (DMEM) medium supplemented with 10% fetal bovine serum, penicillin (50 IU/mL), and streptomycin sulphate (50 μg/mL). Jurkat T cells were cultured in RPMI 1640 medium supplemented with 10% fetal bovine serum, penicillin (50 IU/mL), and streptomycin sulfate (50 μg/mL).

TALL-104 CD8-positive cytotoxic T lymphocytes, obtained from human acute lymphoblastic leukemia (TALL), were cultured in American type culture collection (ATCC)-formulated Iscove’s modified Dulbecco’s medium. To make the complete growth medium, we added recombinant human IL-2 (100 units/mL), human albumin (2.5 μg/mL), D-mannitol (0.5 μg/mL), and fetal bovine serum to a final concentration of 20% as well as penicillin (50 IU/mL) and streptomycin sulfate (50 μg/mL).

### Primary human samples

A total of five nasopharyngeal swabs collected from individuals in May to June 2020 (non-hospitalized patients) infected with SARS-CoV-2 were included into this study. Laboratory confirmation of SARS-CoV-2 was performed at the North Kent Pathology Service (Darent Valley Hospital, Dartford and Gravesham NHS Trust) using reverse transcriptase–polymerase chain reaction (RT-PCR). Specimens were kept at −80°C to ensure reliable results when used in experiments. The experimental work was performed following informed written consent of the participants under the ethical approval 21/HRA/2577 provided by the NHS Health Research Authority.

### Plasmids and transfections

The following plasmids were used in the study: 1) the human codon optimized Wuhan-Hu-1 isolate (GenBank, MN908947.3) ancestral Spike (S) subcloned into the pCAGGS expression vector, and 2) pCAGGS expression vector without an insert was employed as a control plasmid.

Transfections were performed using FuGENE® HD transfection reagent (Promega) according to the manufacturer’s protocol.

### Western blot analysis

Levels of phospho-S423/S425 Smad-3, galectin-9, and granzyme B were analyzed by Western blot and compared to the amounts of β-actin (protein loading control), as previously described (12). LI-COR goat secondary antibodies conjugated with infrared fluorescent dyes, were used as described in the manufacturer’s protocol for visualization of specific target proteins (LI-COR Odyssey imaging system was employed). Western blot data were quantitatively analyzed using Image Studio software, and values were subsequently normalized against those of β-actin named as “actin” in the figures.

### qRT-PCR analysis

To detect PD-L1, CD3ϵ, interferon-γ (IFN-γ), HIF-1α, and vascular endothelial growth factor (VEGF) mRNA levels, we used quantitative real-time PCR (qRT-PCR) (6, 12). Total RNA was isolated using a GenElute™ mammalian total RNA preparation kit (Sigma-Aldrich) according to the manufacturer’s instructions, followed by RT-PCR of a target protein mRNA (also performed according to the manufacturer’s protocol). This was followed by qRT-PCR. The following primers were used:

PD-L1: (forward) 5′-AAATGGAACCTGGCGAAAGC-3′ and (backward) 5′-GATGAGCCCCTCAGGCATTT-3′; CD3ϵ: (forward) 5′-TCCCAACCCAGACTATGAGC-3′ and (backward) 5′-CAAGACTAGCCCAGGAAACAG-3′; IFN-γ: (forward) 5′- TCCCATGGGTTGTGTGTTTA-3′ and (backward) 5′-AAGCACCAGGCATGAAATCT-3′; HIF-1α: (forward) 5′-CTCAAAGTCGGACAGCCTCA-3′ and (backward) 5′-CCCTGCAGTAGGTTTCTGCT-3′; VEGF: (forward) 5′-GTATAAGTCCTGGAGCGT-3′ and (backward) 5′-CTCGGAGGGAGTCCCAAA-3′; and β-actin: (forward) 5′-TGACGGGGTCACCCACACTGTGCCCATCTA-3′ and (backward) 5′-CTAGAAGCATTTGCGGTCGACGATGGAGGG-3′. Reactions were performed using a LightCycler^®^ 480 qRT-PCR machine and SYBR Green I Master kit (Roche, Burgess Hill, UK). The assay was performed according to the manufacturer’s protocol. Values of investigated protein mRNA levels were normalized against those of β-actin.

### Chromatin immunoprecipitation

Chromatin immunoprecipitation (ChIP) was performed as described recently (12, 13). In brief, 5 × 10^6^ cells were subjected to immunoprecipitation. Cross-linking was then performed using 1.42% formaldehyde, followed by quenching for 5 min with 125 mM glycine. Cells were then washed twice with Phosphate-buffered saline (PBS) and subjected to ChIP according to ChIP-IT high-sensitivity kit (Active Motif) protocol. Immunoprecipitation was performed using anti–HIF-1α antibody. IgG isotype control antibody was used for a negative control IP. The epitopes recognized by these antibodies do not overlap with DNA and co-activator binding sites of these proteins. Immunoprecipitated DNA was then purified and subjected to qRT-PCR which was performed as outlined above. The following primers were designed using NCBI Primer-Blast primer designing tool (https://www.ncbi.nlm.nih.gov/tools/primer-blast/): TGF-β: (forward) 5′-ATCGCCATCATCATCATCCACTGAGC-3′ and (backward) 5′-TTGCAGTCCATGGCATAGGG-3′. These primers allow amplification of the fragments of promoter regions of corresponding genes, which surround HIF-1-binding sites [so-called hypoxia response elements (HREs)].

### Enzyme-linked immunosorbent assays

Levels of secreted/soluble galectin-9, TGF-β, and IL-2 were measured in nasal secretion from the back of the nose and throat (nasopharyngeal swab) or cell culture conditioned media, and PD-L1 quantities were also analyzed in cell lysates by ELISA using R&D Systems kits (DY2045 for galectin-9, DY240 for TGF-β, DY202 for IL-2, and DY156 for PD-L1) according to the manufacturer’s protocols.

### On-cell Western analysis

Cell surface levels of RAGE protein were measured using on-cell Western analysis performed using a LI-COR Odyssey imager, and the assay was performed in line with the manufacturer’s recommendations as previously described (14).

### Analysis of HIF-1α PHD and HIF-1 DNA-binding activity

HIF-1α prolyl hydroxylase (PHD) activity was analyzed as described before using the method based on ability of HIF-1α PHD to hydroxylate specific peptide (15). HIF-1 DNA-binding activity was measured by the method, which was described recently (7). Briefly, 96-well Maxisorp™ microtiter plates were coated with streptavidin and blocked with Bovine serum albumin (BSA). A volume of 2 pmol/well biotinylated 2HRE (HRE) containing oligonucleotide was immobilized by 1 h of incubation at room temperature. The plates were then washed with TBST buffer [10 mM Tris-HCl (pH 8.0), 150 mM NaCl, and 0.05% Tween-20), followed by 1 h of incubation with cell lysate at room temperature. The plate was again washed with Tris-buffered saline with Tween 20 (TBST) buffer and mouse anti–HIF-1α antibody (1:1,000 in TBS with 2% BSA) was added. After 1 h of incubation at room temperature, the plate was washed with TBST buffer and then incubated with LI-COR goat anti-mouse secondary antibody labeled with infrared fluorescent dye. After extensive washing with TBST, the plate was scanned using a LI-COR Odyssey fluoroimager.

### Detection of LDHA activity using 2-OG as a substrate and measurement of 2-OH-glutarate

Lactate dehydrogenase A (LDHA) activity was analysed using Sigma-Aldrich assay kit and reagents (with modification - 2-OG was employed as a substrate) according to the standard protocol based on oxidation of NADH+H+. FX11 LDHA inhibitor was used to confirm specificity of the reaction. 2-Hydroxy-glutarate (2-OH-G, D form) was measured using Abcam colorimetric assay kit (ab211070) according to the manufacturer’s protocol.

### Granzyme B activity assays

Granzyme B activity in cell lysates was measured using a fluorometric assay based on the ability of this enzyme to cleave the fluorogenic substrate Ac-IEPD-AFC (Sigma-Aldrich) as described before (16).

### Detection of LKU and IDO-1 activity

L-kynurenine (LKU) levels were analyzed on the basis of ability of this amino acid to react with 4-dimethylamino)-benzaldehyde (17). Briefly, we took 160 µL of cell culture conditioned medium or nasal secretion from the back of the nose and throat (nasopharyngeal swab) and added 10 µL of 30% (v/v) trichloroacetic acid to each sample. Samples were incubated for 30 min at 50°C in order to hydrolyze N-formylkynurenine to L-kynurenine (LKU). The samples were then centrifuged at 3,000 g for 10 min. Supernatants (100 µL) were transferred to wells of a 96-well flat-bottom plate and mixed with 100 µL of freshly prepared Ehrlich’s reagent [1.2% w/v 4-(dimethylamino)-benzaldehyde in glacial acetic acid] followed for 10 min of incubation at room temperature. Absorbance was measured using a microplate reader at 492 nm.

IDO-1 activity (or enzymatic conversion of L-Trp into N-formylkynurenine), which was then further converted into LKU, was measured using a previously described method (17) with minor modifications. Briefly, the cell or tissue lysate was added to the reaction mixture containing 50 mM potassium phosphate buffer (pH 6.5), 20 mM ascorbate, catalase (100μg/mL), and 2 mM L-Trp. The reaction was carried out at 37°C for 60 min and terminated by adding 10 μL of 30% (v/v) trichloroacetic acid to 160 μL of sample. Further operations were performed as previously described.

### Characterization of glycolysis and measurement of MGO levels

Glycolytic degradation of glucose was analyzed using a colorimetric assay as described previously (17, 18). Briefly, the assay was performed using cell lysates and the conversion of glucose into lactate in the absence of oxygen. Cell lysates were incubated for 1 h at 37°C with 1% glucose solution in an anaerobic chamber. Two percent trichloroacetic acid solution was then used to precipitate proteins. This was followed by carbohydrate precipitation using saturated CuSO_4_ solution in combination with Ca(OH)_2_ (at a final concentration of 60 mg/mL). Lactate was then converted into acetaldehyde using concentrated H_2_SO_4_ at 90°C for 1 min and cooled on ice. Acetaldehyde was detected using the veratrole (1,2-dimethoxybenzene) test. Methyl-glyoxal (MGO) was also detected colorimetrically in the cell culture medium following biochemical modifications. In brief, MGO was condensed with reduced Glutathione (GSH) (1 mM) for 10 min at 37°C. The complex was then converted into lactate by glyoxalases I and II at pH 8.0 (glyoxalase I converted the complex into D-lactoyl-glutathione, which was then converted into lactate by glyoxalase II). Lactate was then detected colorimetrically, as described above.

### Analysis of SHP-2–like phosphatase activity

Activity of DiFMUP (6,8-difluoro-4-methylumbelliferyl phosphate)–specific phosphatases (here, we call them “SHP-2–like phosphatases”) was analyzed using a flurometric assay kit (E12020, Thermo Fisher Scientific) according to the manufacturer’s instructions.

### Annexin V/propidium iodide assay and cell proliferation assay

The percentage of apoptotic/necrotic [propidium iodide (PI)–positive)] cells was measured using an Annexin V/PI assay kit (APOAF-20TST, Sigma-Merck). We measured Annexin V and PI-positive cells using flow cytometry according to the manufacturer’s protocol. Quadrant gates were used to distinguish the populations by fluorescence: Annexin V+ PI−, Annexin V+ PI+, and Annexin V− PI+ populations from the double-negative population (Annexin V− PI−). The gate was set according to the unstained control. We also measured Annexin V and PI-positive cells by fluorometric assay using 96-well plates, where the cells could stay adherent in order to rule out false-positive and false-negative results. The results were calculated as % of cells stimulated with 1mM H_2_O_2_ for 6 h. Cell proliferative activity was assessed using an MTS assay kit (Promega, G3580) according to the manufacturer’s protocol.

### Statistical analysis

Each experiment was performed at least four times, and statistical analysis was conducted using a two-tailed Student's *t*-test or the Kruskal-Wallis test. Statistical probabilities (p) were expressed as * when p < 0.05, ** when p < 0.01, and *** when p < 0.001.

## Results

### Wuhan strain of SARS-CoV-2 activates immune evasion machinery in human tissues

**Figure 1:**
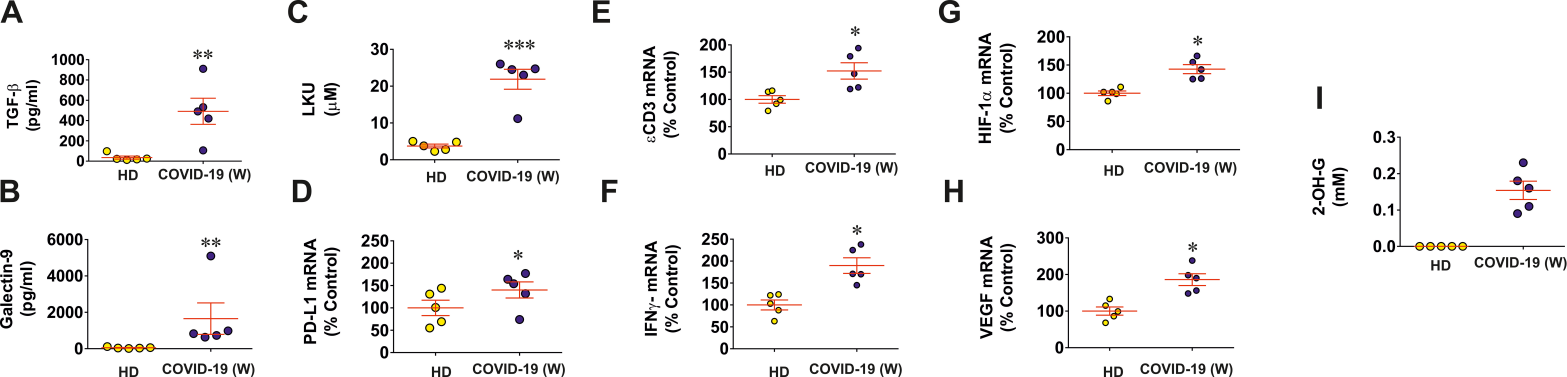
Wuhan strain of SARS-CoV-2 triggers activation of immune evasion machinery in human tissues. Nasopharyngeal swabs of five COVID-19 non-hospitalized patients (infected with Wuhan strain virus) and five healthy individuals were analyzed for specific markers of immune response and evasion as outlined in Materials and Methods. TGF-β **(A)** and galectin-9 **(B)** levels were measured by ELISA; LKU levels **(C)** were analyzed colorimetrically; PD-L1 **(D)**, ϵCD3 **(E)**, IFN-γ **(F)**, HIF-1α **(G)**, and VEGF **(H)** mRNA levels were analyzed using qRT-PCR; 2-OH-G levels were analyzed spectrophotometrically **(I)**. Data are the mean values ± SEM of five independent experiments. * p<0.05; ** p<0.01 and *** p < 0.001 vs HD.

First of all, we investigated nasopharyngeal swabs (taken in the active phase of disease at diagnosis and before treatment) obtained from five non-hospitalized individuals affected by Wuhan strain of SARS-CoV-2 virus versus five healthy individuals. We found that, in swabs of COVID-19 individuals, the levels of galectin-9, TGF-β, and LKU were highly upregulated compared to those of healthy individuals (**Figures 1A–C**). We also observed significant upregulation of PD-L1 mRNA levels in COVID-19 individuals (**Figure 1D**) as well as significant upregulation of ϵCD3 mRNA levels (**Figure 1E**). This means increased levels of infiltrated CD3-positive T lymphocytes, because T cells express and use this protein, and, respectively, a significant upregulation of IFN-γ mRNA levels was observed. Interestingly, mRNA levels of both HIF-1α and VEGF (encoded by HIF-1 downstream gene and, thus, used to analyze HIF-1 activity) were significantly increased in the swabs of COVID-19 individuals (**Figures 1G, H**). This means that HIF-1 transcription complex was more active in COVID-19 individuals because it is known to specifically upregulate VEGF gene transcription (19). However, we were keen to understand the cause of this effect because the tissues subjected to taking the swabs cannot have highly decreased oxygen availability. On the other hand, the S protein of SARS-CoV-2 was reported to upregulate aerobic glycolysis leading to translocation of all the pyruvate into mitochondria (20, 21). This can leave existing lactate dehydrogenase (LDH) A enzyme molecules “unemployed.” In addition, in this case, LDH A can in theory convert cytosolic 2-OG into L-2-OH-G (22). The latter cannot be used as a cofactor in HIF-1α PHD reaction, leading to attenuation of this reaction and stabilization/accumulation of HIF-1α protein.

To investigate this hypotheis of 2-OG conversion/removal, we measured D-2-OH-G in swabs of both SARS-CoV-2–infected and healthy individuals. We found that 2-OH-G was not detectable at all in the swabs of all healthy individuals and was clearly detectable in swabs of COVID-19 individuals (**Figure 1I**). As such, we hypothesized that this process triggers activation of HIF-1, which was shown to upregulate TGF-β production (7), and TGF-β is known to induce expression of galectin-9, PD-L1, and IDO1 (converts amino acid L-tryptophan into formyl-kynurenine, which is then turned into LKU) via the Smad3 pathway (6, 17). Thus, galectin-9, PD-L1, and LKU are suppressing T-cell function (6, 17).

### S protein of SARS-CoV-2 (Wuhan strain) triggers activation of galectin-9, PD-L1, and IDO-1/LKU immune evasion pathways via hypoxic signaling and TGF-β

**Figure 2:**
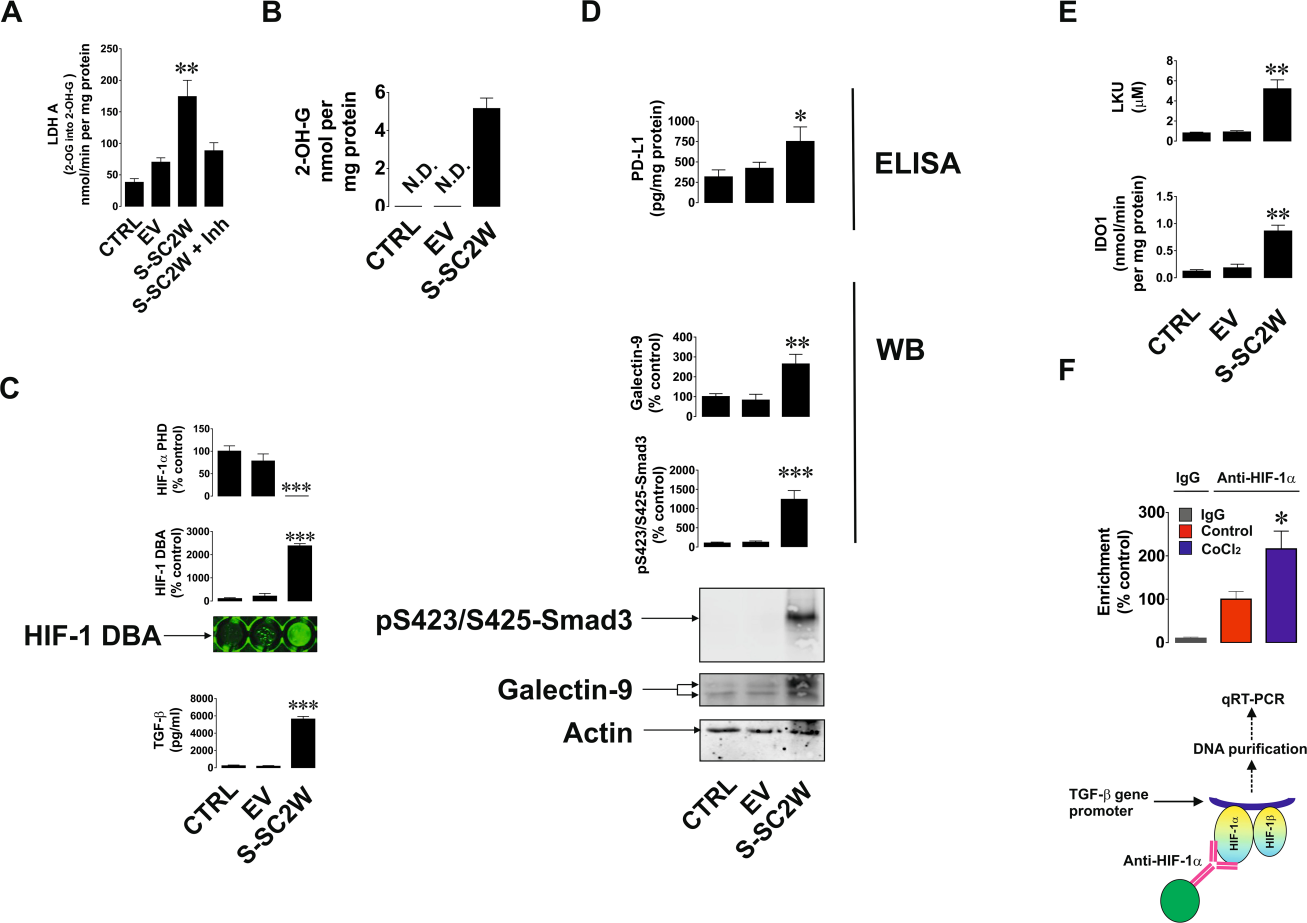
S protein of Wuhan strain of SARS-CoV-2 triggers activation of immune evasion machinery in human bronchial epithelial cells. BEAS-2B cells were transfected with EV or S (Wuhan strain, abbreviated as S-SC2W) and cultured for 40 h as outlined in Materials and Methods. This was followed by investigation of immune evasion machinery and its activation pathways. Specifically, 2-OH-G levels **(A)** and LDHA 2-OG converting activity **(B)** were analyzed colorimetrically; HIF-1α PHD activity and HIF-1 DNA-binding activity were analyzed as described in Materials and Methods; levels of TGF-β were measured by ELISA **(C)**; levels of p-Smad3 and galectin-9 were analyzed by Western blot, whereas PD-L1 levels were measured by ELISA **(D)**; IDO1 activity and LKU levels were measured as outlined in Materials and Methods **(E)**. Resting BEAS-2B cells and those treated for 4 h with 50 µM CoCl_2_ were subjected to ChIP with anti–HIF-1α antibody followed by RT-PCR for presence of TGF-β gene promoter region fragments **(F)**. Images are from one experiment representative of five, which gave similar results. Quantitative data are the mean values ± SEM of five independent experiments. *p < 0.05; **p < 0.01, and ***p < 0.001 vs. control.

**Figure 3:**
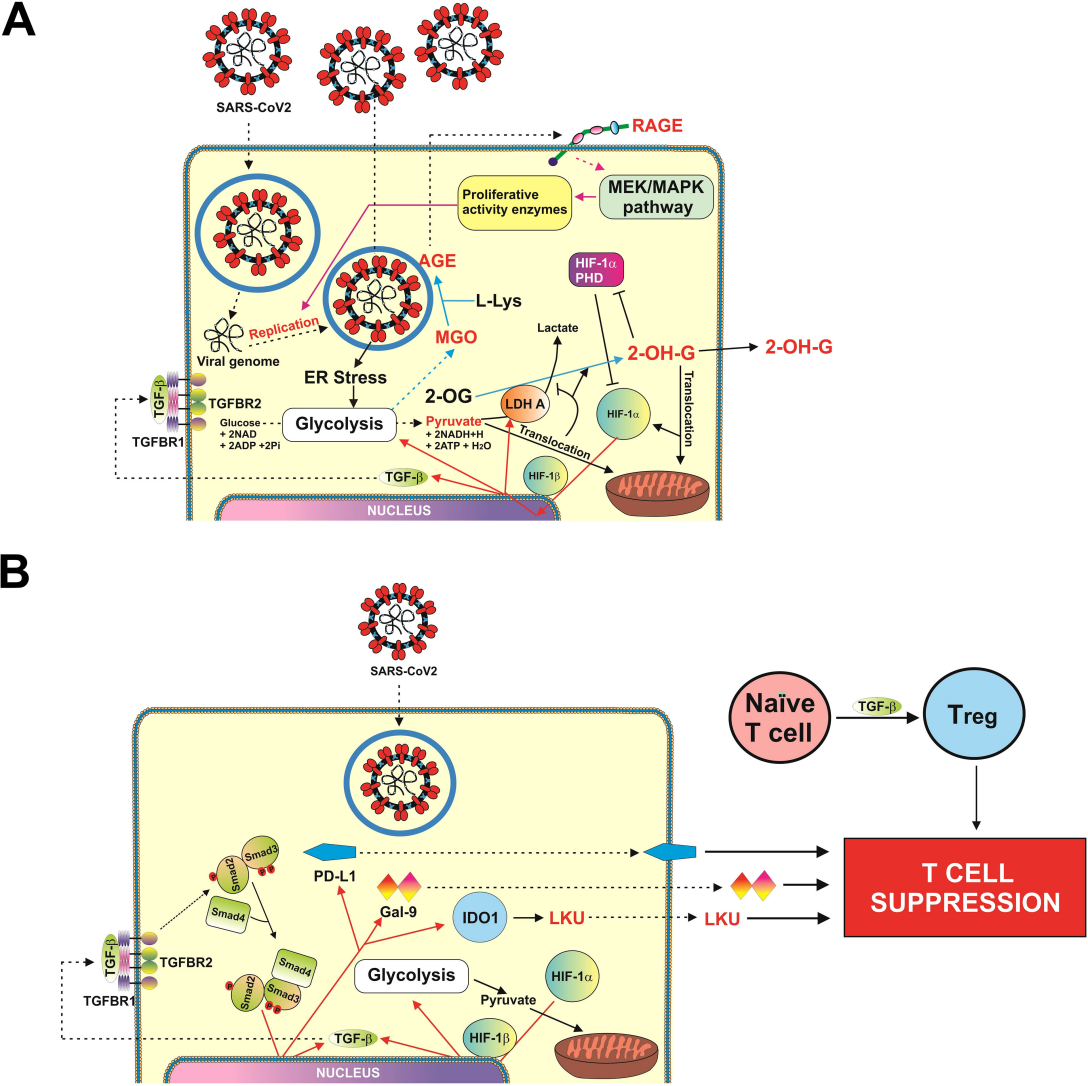
Scheme illustrating possible mechanisms of SARScCoV-2 Wuhan strain-induced activation of HIF-1/TGF-B pathway **(A)**, and SARS-CoV-2 Wuhan strain–dependent TGF-β–induced activation of immune evasion machinery **(B)**.

To verify the finding and hypotheses described above in the *in vitro* cell culture model, we used human bronchial epithelium BEAS-2B cells. Cells were transfected with Wuhan SARS-CoV-2 S protein expression plasmid (S-SC2W) vs. empty vector (EV; control). After transfection cells were cultured for 40 h followed by investigation of the effects caused by S protein presence in the cells. We found that transfection of EV or S-SC2W containing vector reduced cell proliferative activity of BEAS-2B cells but did not affect their viability (**Supplementary Figures 1A–C**). We also found that 2-OH-G was not detectable in wild-type BEAS-2B cells as well as in the cells transfected with EV. But in the cells transfected with S-SC2W, it was clearly detectable (**Figure 2A**). At the same time, we were measuring LDHA activity using 2-OG as a substrate. It was obviously higher in S protein-containing BEAS-2B cells. At the same time, when LDHA inhibitor was added to the system containing S-SC2W–transfected cell lysate as enzyme preparation, conversion of 2-OG was significantly downregulated confirming the ability of LDHA to act on 2-OG (**Figure 2B**). This led to attenuation of HIF-1α PHD activity as well as high upregulation of HIF-1 DNA-binding activity and TGF-β production by BEAS-2B cells (**Figure 2C**). As a result, glycolysis was significantly upregulated in S-containing BEAS-2B cells compared to that in controls. As a side effect of glycolysis upregulation, cells produced increased amounts of MGO, which is known to trigger generation of advanced glycation end products (AGE; act through RAGE, receptor of AGE, present in all the studied cells), which supports the activity of mitogen-activated protein kinase cascades (14, 18), thus supporting expression of viral proteins/capsids (if we are dealing with functional virus). These results are shown in **Supplementary Figure 2**. Phospho-Smad3 was highly upregulated, which was in line with increased expression of galectin-9 and PD-L1 in S-containing BEAS-2B cells (**Figure 2D**). IDO1 activity and respectively LKU release were highly increased in S-SC2W–containing BEAS-2B cells (**Figure 2E**). We also verified the role of HIF-1 in TGF-β expression by performing ChIP using anti–HIF-1α antibody followed by RT-PCR in resting BEAS-2B cells and those exposed for 4 h to 50 µM CoCl_2_ (to induce HIF-1 activity). We confirmed the direct interaction of HIF-1 with TGF-β gene promoter region (**Figure 2F**). As such, we can confirm that Wuhan SARS-CoV-2 S protein upregulates glycolysis, and we uncovered the mechanism underlying this process. This mechanism also leads to activation of immune evasion machinery similar to the one operated by cancer cells. Schemes outlining the details of these events are shown in **Figures 3A, B**.

### SARS-CoV-2 S protein (Wuhan strain) increases immune evasion activity of infected cells

**Figure 4:**
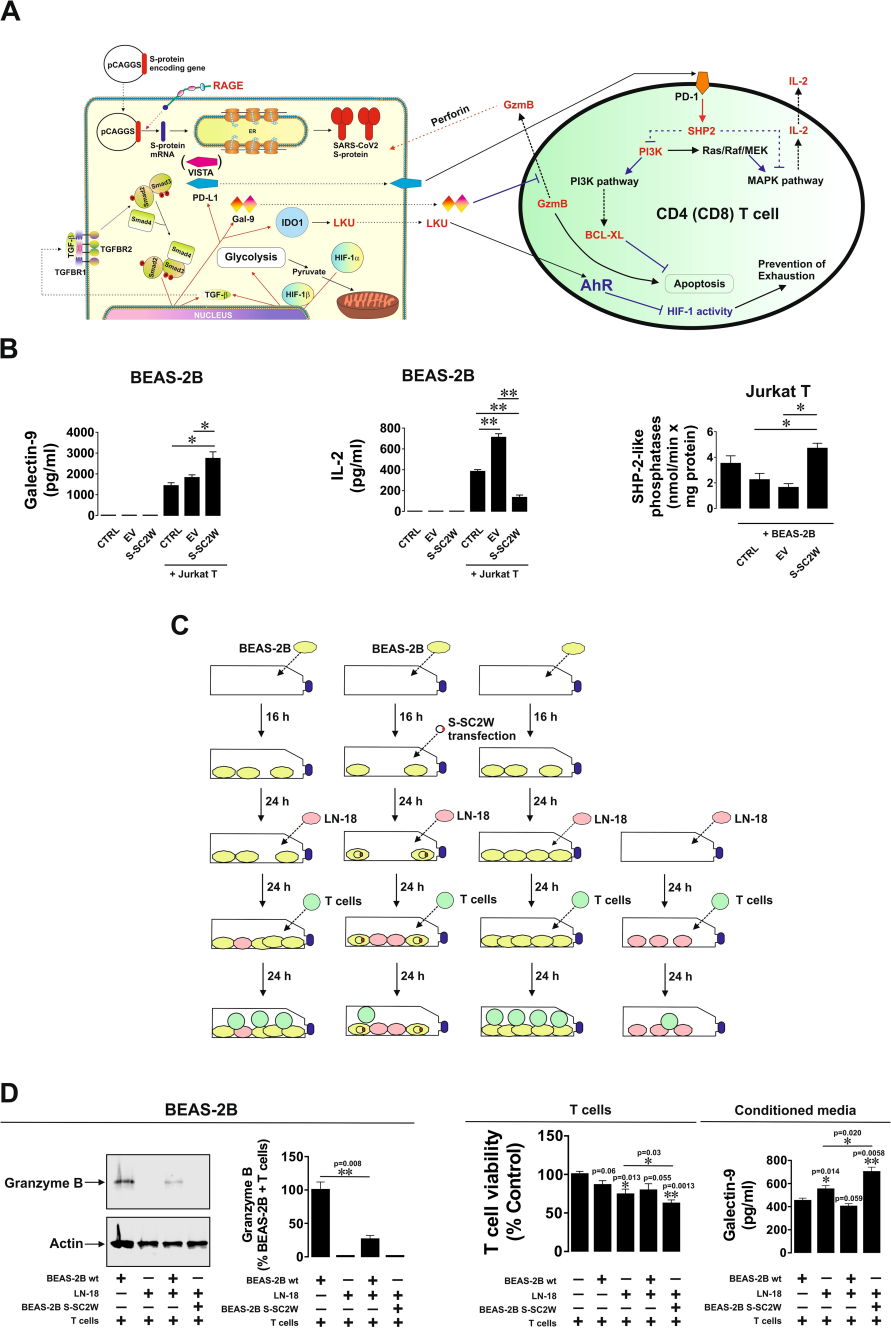
S-SC2W–induced immune evasion machinery suppresses T cell activities in BEAS-2B cells. **(A)** General scheme illustrating how TGF-β–induced immune evasion machinery of BEAS-2B cells can suppress T cell activities. **(B)** BEAS-2B cells transfected with S-SC2W were incubated for 16 h followed by 24 h of co-culturing with Jurkat T cells in the ratio 1:1. Levels of secreted galectin-9 and IL-2 were measured by ELISA in conditioned medium. Activity of SHP-2–like phosphatases was analyzed in Jurkat T cells as outlined in Materials and Methods. **(C)** BEAS-2B cells were plated and cultured for 16 h. Some of the cells were then transfected with S-SC2W, and all the cells were further cultured for 24 h followed by adding equal amounts of LN-18 cells followed by 24 h of co-culture. Then, approximately equal amounts of cytotoxic TALL-104 T cells were added to the co-culture and kept together for the next 24 h. **(D)** Granzyme B presence in adherent cells was assessed using Western blot analysis. Granzyme B activity in these cells was analyzed using fluorometric assay. Viability of T cells was analyzed, and levels of secreted galectin-9 were detected in the conditioned medium. Data are mean values ± SEM of four independent experiments. *p < 0.05 and **p < 0.01 between indicated events.

We asked whether BEAS-2B cells transfected with SARS-CoV-2 S protein (Wuhan strain) display stronger immune evasion activity compared to wild-type BEAS-2B cells. We co-cultured wild-type BEAS-2B cells and those transfected with EV or S-SC2W with Jurkat T cells (as T helpers) at the ratio 1:1 for 16 h. BEAS-2B cells are expressing MHC II type proteins and, thus, can engage in interactions with CD4-positive T helpers (23). We found that, in the co-culture containing S-SC2W–transfected BEAS-2B cells, levels of released galectin-9 were significantly higher and levels of IL-2 were significantly lower compared to those in other cell types (**Figures 4A, B**). At the same time, in Jurkat T cells co-cultured with BEAS-2B containing S protein, activities of SHP-2–like phosphatases were clearly upregulated, altogether meaning activation of PD-1-type signaling events (24). BEAS-2B cells (wild type and those transfected with S-SC2W) were co-cultured with LN-18 high-grade glioblastoma cells in order to model co-habitation of cancer and SARS-CoV-2–affected cells. Then, TALL-104 cytotoxic T cells were added in the approximate ratio 1:1 (**Figure 4C**) for 24 h. We found that, in the case where we had co-habitation of LN-18 and S-SC2W–transfected BEAS-2B cells, injection of granzyme B into adherent cells was completely attenuated compared to other cases (**Figures 4C, D**). At the same time, in this co-culture, there was the highest level of T cell killing and the highest level of galectin-9 secretion. These results suggest that S-SC2W–transfected cells support cancer cells in their fight with cytotoxic T cells *in vitro*. To further verify the immunosuppressive activity of BEAS-2B cells transfected with S-SC2W, we performed the same experiment (the outline is shown in **Supplementary Figure 3A**) but co-culturing just BEAS-2B cells (wild type, BEAS-2B transfected with EV, or BEAS-2B transfected with S-SC2W) with TALL-104 cytotoxic T cells as described above. BEAS-2B transfected with S-SC2W showed similar immune evasion potential as reported in **Figure 4** (see **Supplementary Figure 3B** for details). In addition, it was much higher compared to wild-type BEAS-2B cells and those transfected with EV (**Supplementary Figure 3B**).

## Discussion

There has been an increasing concern regarding Wuhan strain of SARS-CoV-2 being highly immune evasive. In our work, we have demonstrated that this virus manages to trigger a complex pathway leading to activation of HIF-1 transcription complex. This was found to be triggered specifically by S protein, which is in line with previous observations on S protein–dependent upregulation of glycolysis, the process, which is downstream of HIF-1 signaling (20, 21). By activating energy metabolism, which is required for virus replication, it manages to make LDHA and possibly other enzymes to convert cellular 2-OG into 2-OH-G (**Figures 1I**, **2A, B**). Inrerestingly, a similar pathway was reported for different cancers (e.g., acute myeloid leukaemia), where mutated form of isocitrate-dehydrogenase type 1 converts cellular 2-OG into 2-OH-G (25). Most likely, SARS-CoV-2 S protein manages to trigger this reaction just by reorganizing cellular energy metabolism. It was reported previously that S protein of different coronaviruses including SARS-CoV, which caused an outbreak in 2003, induces heavy endoplasmic reticulum stress (26, 27). SARS-CoV-2 is not an exception and was also reported to trigger severe ER stress (28). This kind of reaction is likely to promote aerobic glycolysis, thus leading to generation of pyruvate which is then translocated into mitochondria (29). As such, cytosolic LDHA could potentially get involved in acting on other substrates like 2-OG, converting it into 2-OH-G. However, one cannot rule out a possibility of potential involvement of other energy metabolism enzymes in this process (especially in generation of D-2-OH-G) because ER stress induced by SARS-CoV-2 and even by its S protein will significantly impact cellular energy metabolism. Generated 2-OH-G cannot be used as a co-factor in HIF-1α PHD reaction, which required 2-OG. PHD reaction (2-OG–dependent) would normally lead to degradation of HIF-1α protein (15). As it does not take place, this makes HIF-1 transcription complex highly active. We observed this activation both *in vivo* (upregulation of HIF-1α mRNA levels and VEGF transcription, **Figures 1G, H**) and *in vitro* (activation of glycolysis, **Supplementary Figure 2**). Active HIF-1 triggers expression of TGF-β, as observed previously (7) and confirmed in our study using ChIP assay (**Figure 2F**). TGF-β displays autocrine activity and, through Smad3 pathway, induces activation of immune checkpoint proteins—galectin-9, PD-L1, and IDO1, the enzyme that is directly involved in production of LKU (13, 17). These processes are schematically illustrated in **Figure 3**. Interestingly, we also observed upregulation of IFN-γ mRNA levels in swabs of COVID-19 patients (**Figure 1F**). As we have recently reported, both TGF-β and IFN-γ are capable of inducing IDO1 activity, leading to increased LKU production (17). As it was shown before, all the immune checkpoints discussed here impair activities of T cells, leading to their exhaustion and programmed death (mainly cytotoxic T cells) (6, 13, 16, 17). At the same time, it is important to mention that upregulated glycolysis leads to increased MGO production (**Supplementary Figure 2**). Biochemically, this process also can work in favor of the virus because MGO engages in generation of AGE products, which can display autocrine activity and act through RAGE. This leads to activation of mitogenic pathways (such as mitogen-activated protein kinase cascades) (30, 31), which, in the case of infection, might well support replication of the virus inside the infected cell.

As we can see from our experiments, this immune evasion machinery is effective against helper activity of Jurkat T cells (**Figures 4A, B**). At the same time, co-habitation of cells transfected with S-SC2W with brain cancer cells obviously supports LN-18 high-grade glioblastoma cells in their fight with TALL-104 cytotoxic T cells by contributing their immune evasion machinery to the one used by cancer cells (**Figures 4C, D**). This suggests that S-SC2W–transfected cells actively support immune evasion pathways of cancer cells, which they are co-cultured/co-habit with.

As such, it is evident that, if malignant cells co-habit with cancer cells in the same tissue, then they may support cancer immune evasion machinery and, thus, can promote growth of malignant tumor and cancer progression (schematically, this is illustrated in **Supplementary Figure 4**). It would be really important to study whether SARS-CoV-2 infects malignant cells *in vivo* and, thus, could influence immunosuppressive tumor microenvironment. Our work has demonstrated that SARS-CoV-2 (Wuhan strain) can activate the immune evasion pathways normally used by cancer cells. From this work, one could see that SARS-CoV-2 could be a potential promoter of cancer progression, which would support and enhance the immune evasion machinery. However, the question if SARS-CoV-2 can actually trigger the process of malignant transformation remains to be addressed in the future. Our work highlights the importance of consideration to apply different approaches, which would allow to downregulate TGF-β signaling in COVID-19 patients in order to reduce the immune evasion effects of the virus and also importance of vaccination in order to neutralise the virus and prevent it from entering target cells and causing the effects described above.

Taken together, our results demonstrated, for the first time, that S protein of the Wuhan strain of SARS-CoV-2 activates immune evasion machinery similar to the one operated by cancer cells and, thus, can in theory support development of malignancies. This work suggests possible novel targets for managing COVID-19 and long COVID symptoms to prevent activation of immune evasion machinery. Other SARS-CoV-2 variants need to be investigated to confirm similar immune evasive effects and activities and, thus, verify if the observed effects are common for all types of COVID-19 infection or just specific to Wuhan strain.

## Data Availability

The datasets used and/or analysed during the current study are available from corresponding authors on reasonable request.
