# Supplementary material for: Wuhan strain of SARS-CoV-2 triggers activation of immune evasion machinery similar to the one operated by cancer cells
Source: Front Immunol. 2025 Jun 18;16:1599352. doi: 10.3389/fimmu.2025.1599352 (PMC12213721; doi:10.3389/fimmu.2025.1599352)
Supplement: Supplementary file 1 [file DataSheet1.docx]

**SUPPLEMENTARY INFORMATION**

**Wuhan strain of SARS-CoV-2 triggers activation of immune evasion machinery similar to the one operated by cancer cells**

**Maryam Abooali, Inna M. Yasinska, Gauri Thapa, Xi Lei, Kelly da Costa, Stephanie Schlichtner, Steffen M. Berger, Elizaveta Fasler-Kan, Nigel J. Temperton, Romina Vuono and Vadim V. Sumbayev**


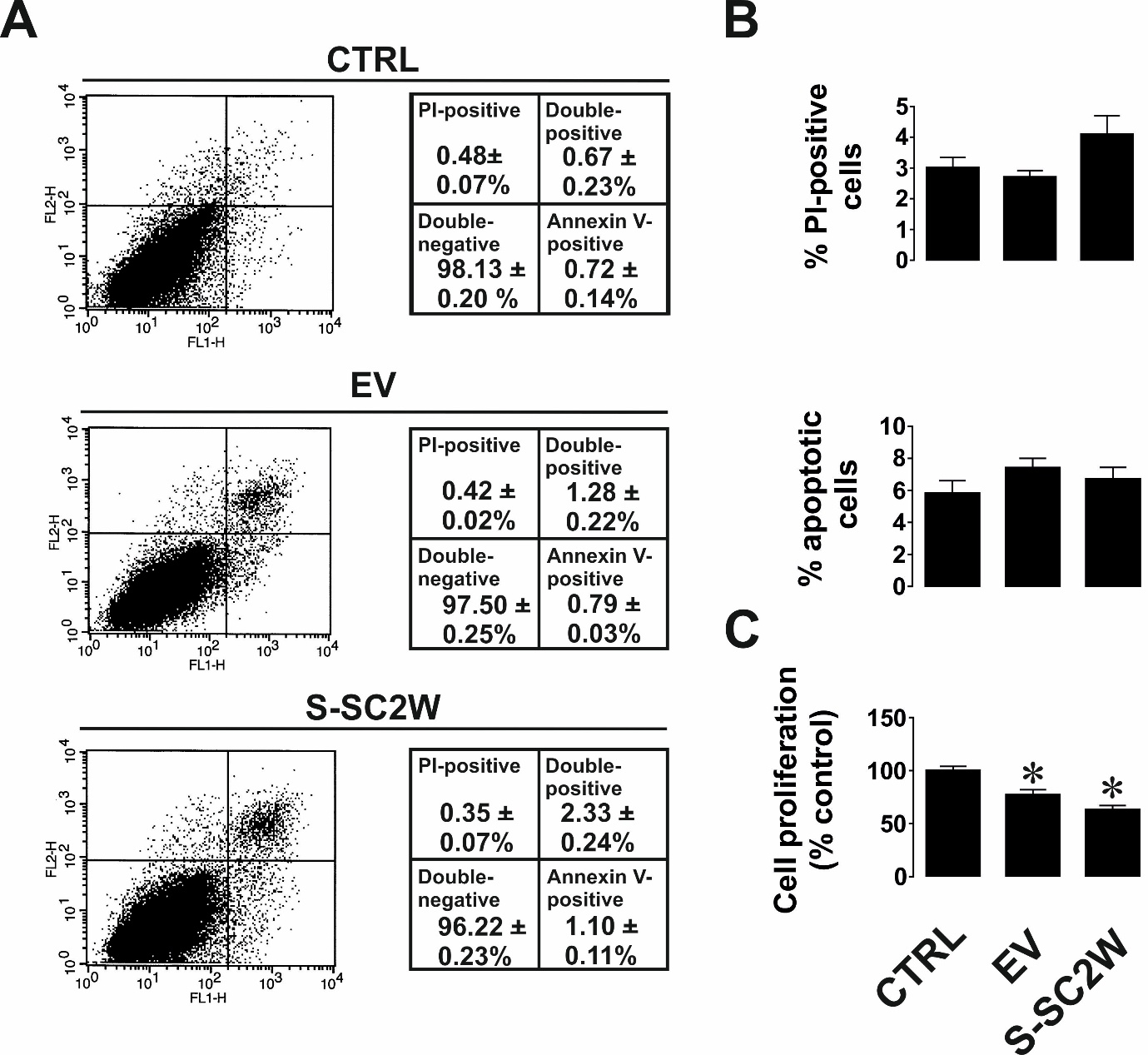


**Supplementary figure 1. SARS-CoV-2 Wuhan S protein (S-SC2W) downregulates proliferative activity but does not affect viability of BEAS-2B cells.** BEAS-2B cells were transfected with EV or S-SC2W and incubated for 40 h followed by measurement of PI-positive (necrotic/late apoptotic cells) and annexin V-positive (apoptotic cells) using **(A)** flow cytometry and **(B)** fluorometric assay. **(C)** MTS test was performed to assess cell proliferative activity. Data are mean values ± SEM of 5 independent experiments. * - p<0.05 vs control**.**


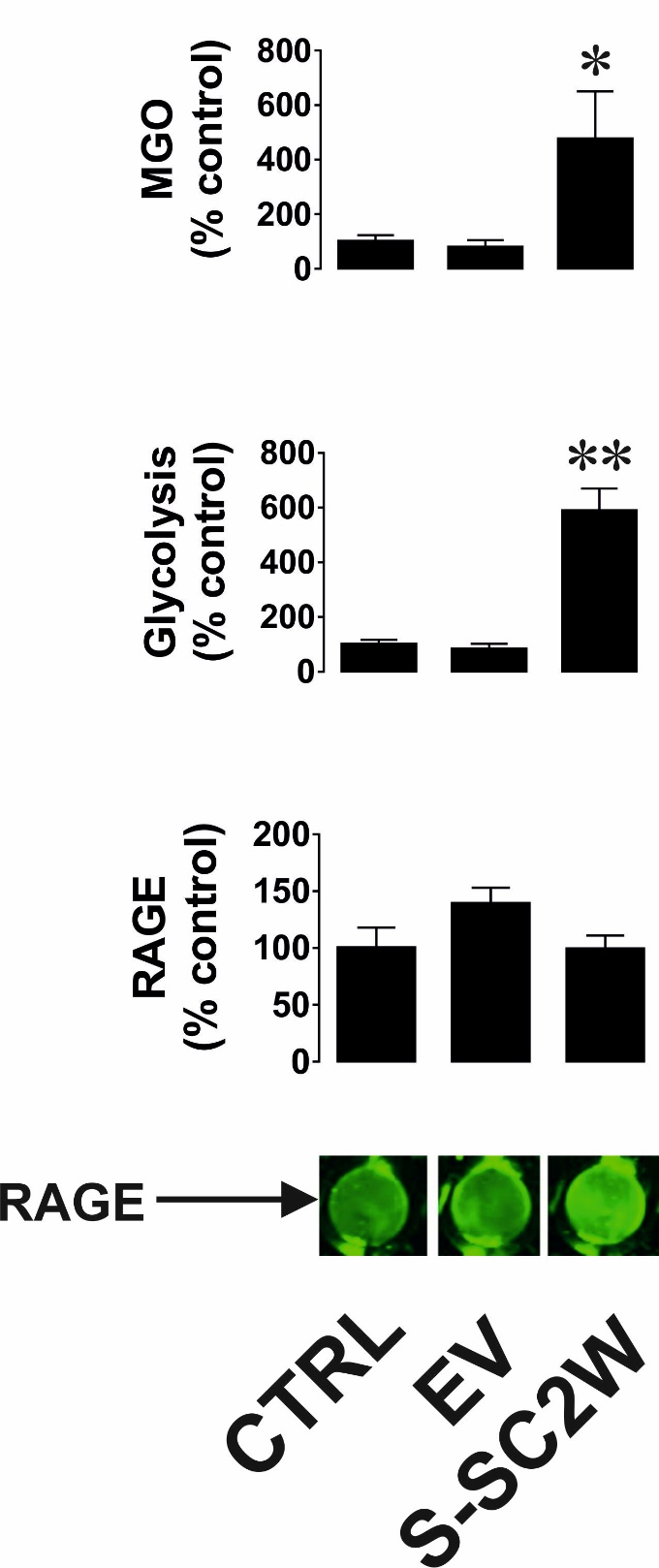


**Supplementary figure 2. SARS-CoV-2 Wuhan S protein (S-SC2W) upregulates glycolysis and MGO production and does not affect RAGE surface presence in BEAS-2B cells.** BEAS-2B cells were transfected with EV or S-SC2W and incubated for 40 h followed by characterisation of glycolysis, detection of MGO production and RAGE surface presence as outlined in Materials and Methods. Data are mean values ± SEM of 5 independent experiments. * - p<0.05 and ** - p< 0.01 vs control**.**

**
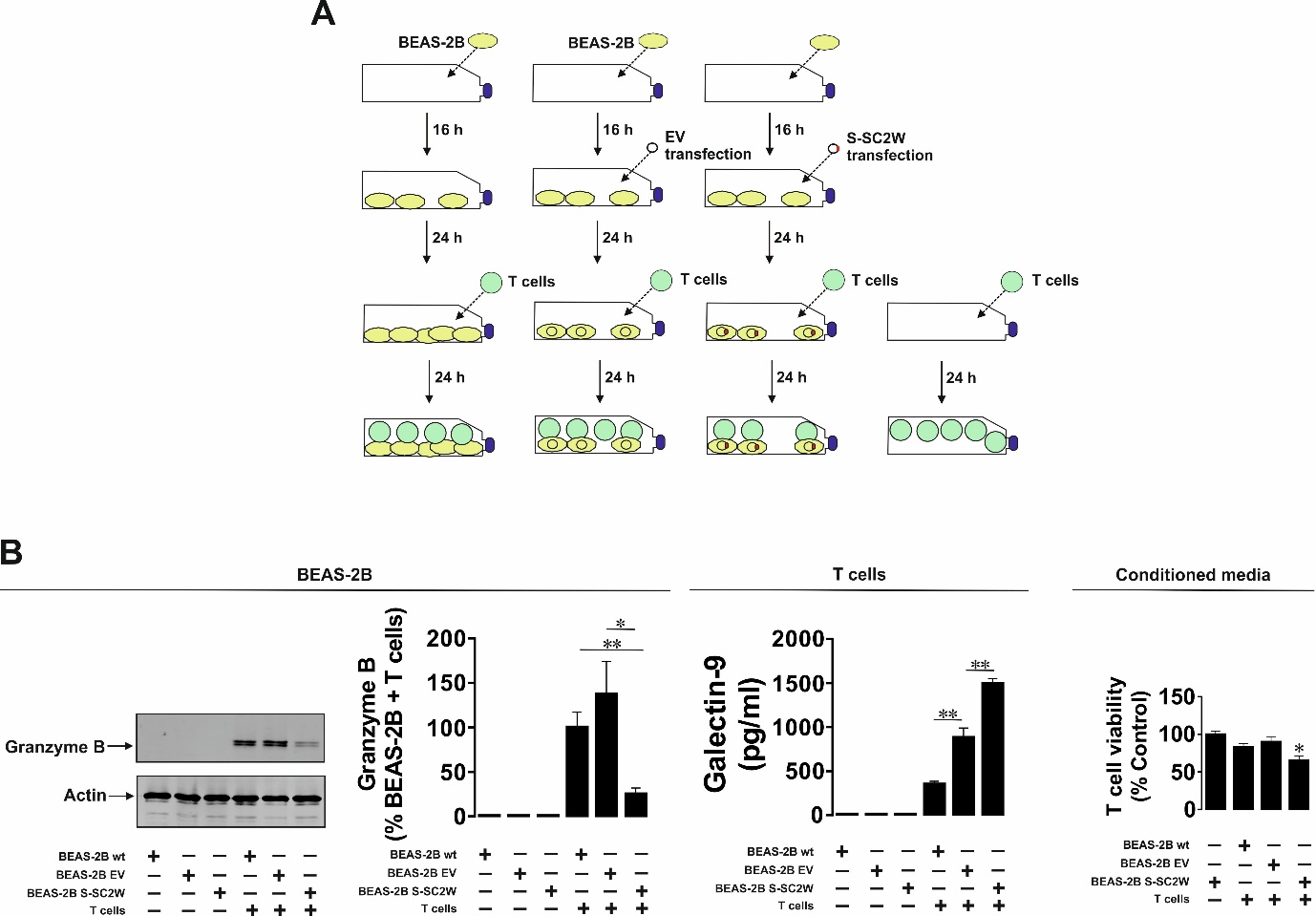
**

**Supplementary figure 3. S-SC2W-induced immune evasion machinery suppresses T cell activities in BEAS-2B cells. (A)** BEAS-2B cells were plated and cultured for 16 h. Some of the cells were then transfected with empty vector or S-SC2W and all the cells were further cultured for 24 h.Then, approximately equal amounts of cytotoxic TALL-104 T cells were added to the co-culture and kept together for the next 24 h. **(B)** Granzyme B presence in adherent cells was assessed using Western blot analysis. Granzyme B activity in these cells was analysed using fluorometric assay. Viability of T cells was analysed and levels of secreted galectin-9 were detected in the conditioned medium. Data are mean values ± SEM of 4 independent experiments. * - p<0.05 and ** - p< 0.01 between indicated events.

**
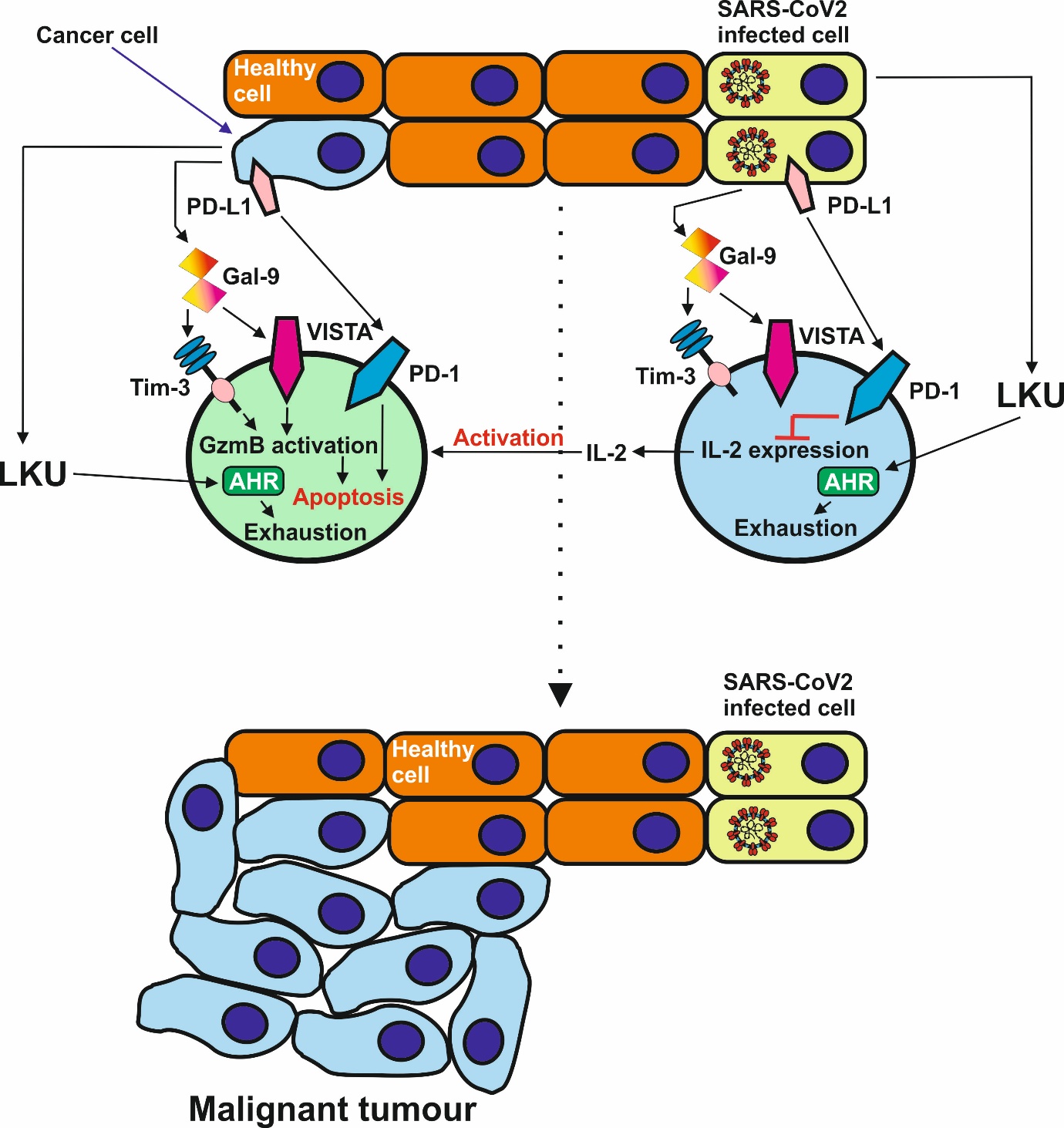
**

**Supplementary figure 4. Scheme illustrating possible pathways supporting cancer immune evasion machinery of malignant cells by Wuhan strain of SARS-CoV2 infecting healthy cells in a tissue affected by malignant transformation.**
